# No increased mortality after total hip arthroplasty in patients with a history of pediatric hip disease: a matched, population-based cohort study on 4,043 patients

**DOI:** 10.1080/17453674.2021.1963582

**Published:** 2021-08-16

**Authors:** Miriam G Wadström, Nils P Hailer, Yasmin D Hailer

**Affiliations:** Section of Orthopedics, Department of Surgical Sciences, Uppsala University, Sweden

## Abstract

Background and purpose — Patients with pediatric hip diseases are more comorbid than the general population and at risk of premature, secondary osteoarthritis, often leading to total hip arthroplasty (THA). We investigated whether THA confers an increased mortality in this cohort.

Patients and methods — We identified 4,043 patients with a history of Legg–Calvé–Perthes disease (LCPD), slipped capital femoral epiphysis (SCFE), or developmental dysplasia of the hip (DDH) in the Swedish Hip Arthroplasty Register (SHAR) between 1992 and 2012. For each patient, we matched 5 controls from the general population for age, sex, and place of residence, and acquired information on all participants’ socioeconomic background and comorbidities. Mortality after THA was estimated according to Kaplan–Meier, and Cox proportional hazard models were fitted to estimate adjusted hazard ratios (HRs) for the risk of death.

Results — Compared with unexposed individuals, patients exposed to a THA due to pediatric hip disease had lower incomes, lower educational levels, and a higher degree of comorbidity but a statistically non-significant attenuation of 90-day mortality (HR 0.9; 95% CI 0.4–2.0) and a lower risk of overall mortality (HR 0.8; CI 0.7–0.9).

Interpretation — Patients exposed to THA due to a history of pediatric hip disease have a slightly lower mortality than unexposed individuals. THA seems not to confer increased mortality risks, even in these specific patients with numerous risk factors.

Altered morphology of the hip joint due to pediatric hip diseases, e.g., Legg–Calvé–Perthes disease (LCPD), slipped capital femoral epiphysis (SCFE), or developmental dysplasia of the hip (DDH) is closely linked to early-onset, secondary osteoarthritis (OA) (Jacobsen and Sonne-Holm 2005, Pun 2016) which may lead to total hip arthroplasty (THA) at a young age (Froberg et al. 2011). Thus, the mean age at THA surgery in patients with a history of pediatric hip disease ranges from 38 to 55 years (Traina et al. 2011, Engesaeter et al. 2012), whereas it ranges from 65 to 70 years in patients with primary OA (Engesaeter et al. 2012, Fang et al. 2015, Cnudde et al. 2018). Studies from Nordic countries report that between 4% and 9% of all primary THAs are due to pediatric hip disease (Engesaeter et al. 2012).

The long-term outcome and revision rates after THA in patients with previous pediatric hip disease have been studied (Thillemann et al. 2008, Traina et al. 2011), but 90-day mortality and overall mortality after THA in these patients have not yet been investigated. Comorbidities, such as attention deficit hyperactivity disorder (ADHD), depression, cardiovascular disease, hypothyroidism, obesity, and coagulation abnormalities are more common in patients with LCPD and SCFE (Hailer and Nilsson 2014, Perry et al. 2017, Hailer and Hailer 2018, Hailer 2020). In addition, patients with LCPD and SCFE have a higher overall mortality than the general population (Hailer and Nilsson 2014, Hailer 2020). Due to an increased comorbidity burden and possibly increased overall mortality one could therefore fear an increased mortality after THA surgery in these patients.

We therefore investigated whether THA surgery in patients with a pediatric hip disease confers an increased 90-day and overall mortality when compared with the general population.

## Patients and method

### Study design and setting

This population-based longitudinal cohort study includes patients (exposed group) with a history of pediatric hip disease (LCPD, SCFE, DDH) who had unilateral or bilateral THA surgery between 1992 and 2012 and were registered in the SHAR (see below). For each patient, 5 matched controls from the general population were randomly selected based on age, sex, and place of residence (unexposed group). The matching variables seemed appropriate because these 3 pediatric orthopedic hip diseases have different “target populations.” Concerning age, all 3 pediatric orthopedic hip diseases occur at different ages and times with disease differences that might also affect the outcome variable “death.” Concerning sex, LCPD affects mainly male patients and has an underlying geographical variation with regards to the incidence, whereas DDH is more prevalent in female patients, also with a geographical variation. The sex-ratio of SCFE is quite equal.

Swedish citizens have a personal identity number (PNR), which enables the collection of data in national quality registers, including the Swedish Population Register, with information on the individual’s birth, death, place of residence, and civil status. The Swedish National Patient Register (NPR) collects information about in- and outpatient care using the ICD code system and the Nordic Medico-Statistical Committee (NOMESCO) codes for the classification of treatments and surgical procedures. The NPR was established in 1964 with data limited to inpatient visits. In 1987, it became mandatory to report to the NPR nationwide. Since 2001, the register also covers outpatient care. The quality of the NPR is continuously controlled and improved by the Swedish National Board of Health and Welfare. Studies on the validity of the registry showed a high validity for the inpatient care (Ludvigsson et al. 2011). The Swedish Mortality Database (SMD) obtains information concerning the causes of death for all individuals in Sweden. The Swedish Hip Arthroplasty Register (SHAR) (Söderman 2000) is a national quality register that has been used since 1979. This register includes data on the patient’s age, sex, and diagnosis together with information regarding the surgical technique and type of implant. Patients were identified in the SHAR when exposed to primary THA as a result of either LCPD, SCFE, or DDH.

Variables of interest were collected from the SHAR, NPR, Swedish Population Register, and SMD. Age was divided into 4 groups (<50, 50–59, 60–74, >74 years). The comorbidity level was expressed by the Charlson Comorbidity Index (CCI) classified into 3 categories (CCI = 0, CCI = 1–2, CCI > 2). Income levels were divided into quartiles, and the level of education was categorized into the 4 levels “less than primary school”, “primary school”, “high school”, or “university level.”

### Statistics

The study includes patients from the beginning of 1992. Follow-up ended on December 31, 2012, emigration, or death, whichever occurred 1st. The endpoint mortality was noted after 1st surgery (exposed group). Continuous variables were described using means with (SD). Counts of categorical data were examined using the chi-square test. We investigated the 90-day and overall mortality of exposed patients compared with unexposed individuals. We used the Cox proportional hazards model rather than logistic regression models in order to account for the temporal progression of the event “death” within the 2 groups (McNutt et al. 2003). The assumption of proportionality was fulfilled, as investigated by visual control of cumulative incidence functions and by calculating Schoenfeld’s residuals. Cumulative unadjusted survival was estimated, and Cox proportional hazards models were fitted to estimate unadjusted and adjusted HRs with 95% confidence intervals (CI) for the risk of death within 90 days or the risk of death during the entire observation period as the outcomes. CCI, levels of income, and education were considered relevant confounders (Garland et al. 2015, Hailer et al. 2016, Weiss et al. 2019). Because the variable “place of residence” had more than 300 levels it was used as a strata variable in the analysis.

Whether or not to adjust for matching variables (age groups, sex, and place of residence at time of surgery) in studies designed such as ours is debated. Both Bland and Altman (1994) and Sjölander and Greenland (2013) recommend adjusting for the matching variables in order to avoid bias in the presence of additional confounders, as in our study. Because we have a large population with access to the Swedish Population register, the number of matching variables is not a limitation in finding appropriate controls, which otherwise can be a disadvantage of matching according to Bland and Altman (1994). Considering this, we found it appropriate to include the matching variables in addition to the confounding variables when estimating the HR, but, as suggested during the review process of this study, we performed a supplementary analysis where HR was estimated after adjustment for confounders but not for matching variables. Here, we found that the obtained HR differed only marginally between models with and without adjustment for the matching variables.

Statistical significance was set at p < 0.05. For analyses, R statistical software, version 3.5.3, package “survival” 2.43-3 and R studio version 1.2.1335 was used (R Foundation for Statistical Computing, Vienna, Austria).

### Ethics, funding, and potential conflicts of interest

Ethical approval for the study was granted by the Regional Ethical Review Board in Gothenburg (reg nr 2013:360/13). This study was supported by a grant based on the ALF agreement (regional funds for participating in the education and training of doctors, implementing clinical research and developing health and medical care) and by a grant from the Swedish Research Council to NPH (VR 2018–02612). The authors report no conflicts of interest.

## Results

### Characteristics of the study population (Table 1)

The study population comprised 23,431 individuals: 4,043 patients with a history of THA surgery due to pediatric hip disease (exposed group), and 19,388 matched controls (unexposed group). Of the exposed group, 6% had a history of LCPD (38% females), 2% had SCFE (44% females), and 92% had DDH (67% females). 974 (24%) also had surgery to the contralateral hip during the observed period. The mean delay between the 1st and 2nd THA was 2.5 years. 72 patients had surgery to both hips within 90 days; 46 patients had 1-stage bilateral THA. No patient who had bilateral THA died within 90 days of surgery, and 47 bilaterally operated patients died during the observation period.

**Table 1.** Characteristics of the population. Values are count (%)

	Unexposed	Exposed
Factor	n = 19,388	n = 4,043
Dead	1,879 (10)	351 (9)
Age		
< 50	7,519 (39)	1,570 (39)
50–59	5,835 (30)	1,220 (30)
60–74	4,758 (25)	989 (25)
≥ 75	1,276 (7)	264 (7)
Female sex	12,488 (64)	2,600 (64)
CCI		
0	18,263 (94)	3662 (91)
1–2	974 (5)	345 (9)
> 2	151 (1)	36 (1)
Income		
1st quarter	4,945 (26)	1,062 (26)
2nd quarter	4,893 (25)	1,047 (26)
3rd quarter	4,869 (25)	987 (24)
4th quarter	4,667 (24)	943 (23)
Education		
None	284 (2)	75 (2)
9 years	5,302 (27)	1,111 (28)
High school	8,387 (43)	1,824 (45)
University	5,415 (28)	1,033 (26)

CCI = Charlson’s Comorbidity Index

64% of the exposed individuals were female. 94% of the unexposed individuals and 91% of the exposed individuals were classified as CCI = 0. The exposed individuals had a higher degree of comorbidities according to the CCI. 6% of unexposed individuals had a CCI of > 1 and 9.5% of the exposed group had a CCI of > 1. In the exposed group the income levels were somewhat lower compared with the unexposed group.

The mean age at the time of THA surgery for patients with unilateral THA was 54 years (SD 13). Patients who had bilateral THA had a mean age at the time for the 1st THA surgery of 53 years (SD 11) and a mean age at the time for the 2nd THA surgery of 56 years (SD 12). The endpoint mortality was noted only after the 1st THA (exposed group). No patient who had bilateral THA died within 90 days after surgery.

### 90-day mortality after THA (Figure 1)

Among the exposed individuals, 8 (0.20%) died within 90 days after THA surgery, as compared with 43 (0.24%) among unexposed individuals. The adjusted HR for death within 90 days after THA surgery was 0.9 (CI 0.4–2.0). Stratified analyses based on the underlying pediatric hip diagnoses (LCPD, SCFE, and DDH) revealed a risk reduction only in patients with DDH; however, this was not statistically significant.

**Figure 1.**
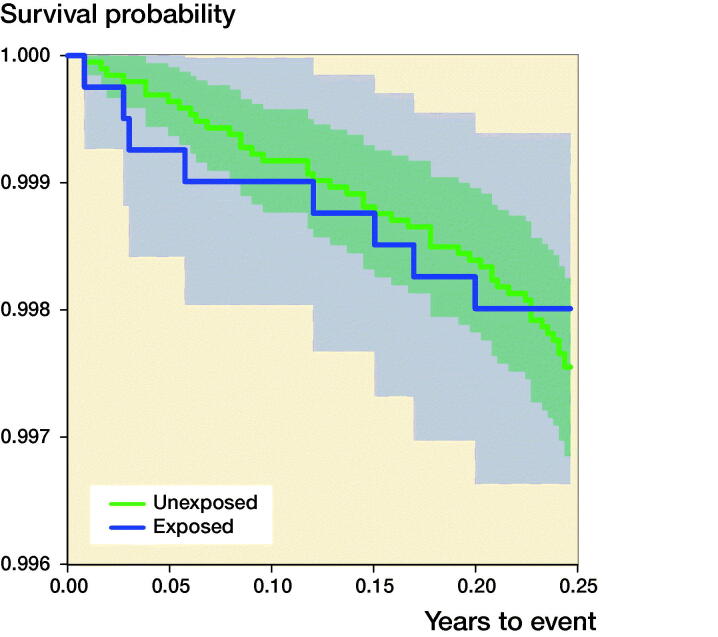
90-day survival analysis for THA patients (exposed, n = 4,043) and controls (unexposed, n = 19,388).

### Risk of overall mortality after THA (Figure 2, Table 2)

351 (8.7%) THA patients died within the entire observation period, compared with 1,879 (9.7%) of the unexposed individuals. Of the deceased THA patients, 29 (8%) had a history of LCPD, 10 (3%) had SCFE, and 312 (89%) had DDH. Patients operated on with a THA as a result of pediatric hip disease had a lower cumulative mortality compared with unexposed controls, and Cox regression analyses indicated an attenuated adjusted HR for overall mortality among the exposed individuals (HR 0.8; CI 0.7–0.9). In the stratified analyses based on the underlying pediatric hip diagnoses (LCPD, SCFE, and DDH), we observed a slightly attenuated overall mortality risk in patients with LCPD and DDH, but statistical significance was seen only in patients with DDH. Patients with a history of SCFE had an adjusted HR of 2.1 (CI 0.8–5.7).

**Table 2.** Overall mortality, adjusted for confounders (CCI, income, and education) and for matching variables (age group, sex, and place of residence)

Item	Crude HR (95% CI)	Adj. HR (95% CI)
All cases	0.9 (0.8–1.0)	0.8 (0.7–0.9)
LCPD	0.8 (0.6–1.3)	0.7 (0.4–1.3)
SCFE	0.9 (0.4–1.7)	2.1 (0.8–5.7)
DDH	0.9 (0.8–1.0)	0.8 (0.7–0.9)

**Figure 2.**
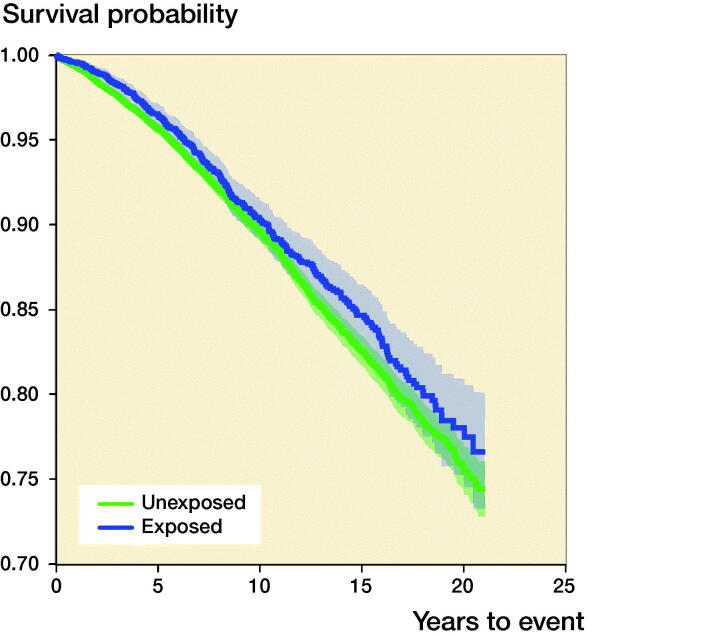
Overall survival analysis for THA patients (exposed, n = 4,043) and controls (unexposed, n = 19,388).

### Causes of death

The most common causes of death in exposed individuals were cardiovascular disease (43%) and cancer (27%), whereas 35% of the unexposed individuals died from cardiovascular disease and 34% from cancer.

## Discussion

In this nationwide cohort study, we found a slightly attenuated 90-day and overall mortality after THA in patients with previous LCPD, SCFE, or DDH, but the attenuation of 90-day mortality was not statistically significant. Despite a higher comorbidity and mortality risk in patients with LCPD and SCFE in general (Hailer and Nilsson 2014, Perry et al. 2017, Hailer and Hailer 2018, Hailer 2020) we found no notable alterations of the mortality risks after THA in such patients, even after stratifying the analyses on the underlying pediatric hip diseases.

We found an 18% lower overall mortality risk in patients who had THA surgery after either LCPD, SCFE, or DDH in childhood when compared with an unexposed sample from the general population. 9% of patients with a history of pediatric hip disease and subsequent THA died during the observation period. This proportion is somewhat higher than in the study by Engesaeter et al. (2012), who investigated a similar patient group and found that 7% of the patients with THA performed for pediatric hip disease died within the study period (1995–2009). In comparison, 18% of their patients with THA because of primary OA died during the study period 1995–2009. The same pattern, with a slightly attenuated mortality risk compared with the general population, has been described in patients who have undergone THA surgery due to primary OA (Lie et al. 2000, Cnudde et al. 2018).

A possible explanation for the lower mortality risk of patient with pediatric hip diseases compared with the general population could be that the majority of the exposed cohort were patients with DDH, in whom risk factors such as those associated with LCPD and SCFE have not been described. However, in our study, we did not see that patients with DDH were less comorbid than the patients with SCFE or LCPD. Further, the stratified analysis based on the underlying pediatric hip diagnosis did not reveal major differences in the mortality risks between these groups. Patients who received THA due to previous pediatric hip disease were relatively young (mean age 53 years) and healthy (94% had a CCI = 0), compared with patients who received THA because of primary OA, who had a median age of 70 years (16–100) and of whom 84% had a low CCI (Weiss et al. 2019). In general, young patients without comorbidities are expected to have a lower risk of mortality, both after surgical interventions and in general. Therefore, we decided to use age- and sex-matched controls from the general population rather than a control group who received THA surgery because of primary OA.

Cardiovascular- and cancer-related deaths were the most prevalent in both groups. Nevertheless, in the group of patients with a pediatric hip disease leading to THA we found a higher proportion of cardiovascular deaths and a lower proportion of cancer-related deaths than in the group of unexposed individuals. The underlying mechanism remains unclear, but a plausible reason could be the increased incidence of cardiovascular diseases in patients with LCPD and SCFE described in previous studies (Hailer et al. 2010, Ucpunar et al. 2018). On the other hand, patients with primary OA of the hip or knee have an increased risk of cardiovascular deaths (e.g., chronic ischemic heart disease and heart failure) (Gordon et al. 2016, Turkiewicz et al. 2019). Cardiovascular disease is closely linked to lower activity levels, and patients with primary OA of the hip or knee often have a reduced level of activity due to pain (Hawker et al. 2014). This could also explain our observations, as pediatric hip disorder leads to early hip pain and subsequently physical inactivity early in life.

### Strength and limitations

This is the first study investigating 90-day and overall mortality risk in patients with a history of pediatric hip diseases and THA surgery. A major strength is the large number of included participants and the nationwide observational design. But, like all registry-based studies, this study has many limitations, including uncertain quality of input data and various types of bias (e.g., selection, detection, and observational bias). The quality of the Swedish registers used in this study is generally good. The SHAR has been validated by Södermann (2000), showing 100% coverage and 95% completeness. Yet, the SHAR has not been validated for secondary OA as a consequence of pediatric hip disease. When registering this specific cause of OA the orthopedic surgeon most likely diagnosed the condition based on radiographs rather than medical journals, and it seems likely that the rate of false-positive pediatric hip diagnoses should be rather low, whereas false-negatives may be more common, i.e., patients classified as having primary OA instead of secondary OA due to a pediatric hip disease could be more common.

The validity of the SMD also needs to be considered. According to the Swedish National Board of Health and Welfare the correctness of reported causes of death is highest in patients with malignant tumors, ischemic heart disease, and in people who died young. The cause of death in the elderly is not classified as accurately because of the difficulty in determining the exact cause of death in patients with many comorbidities. Furthermore, the physician’s external examination often determines the cause of death and an autopsy is seldom performed (Johansson et al. 2009).

Most patients in our exposed cohort were female and had DDH; neither of these factors is known to be related to increased mortality. One factor that might have affected our results is that the patients selected for THA in the group with previous pediatric hip disease might be in good health because of regular contacts with the healthcare system since childhood. In addition, sicker patients are more commonly excluded from surgery, and we do not know to what extent such biases might have impacted our results. The option of selecting patients with primary OA as a control group instead of selecting controls from the general population can be reasonably argued. However, patients with primary OA are generally older than patients with secondary OA after a history of pediatric hip disease. We therefore decided to have a matched control group representing the general population to more clearly define the effect of THA and risk of mortality in patients with a history of pediatric hip disease.

### Conclusion

THA in patients with a pediatric hip disease exposed seems not to confer increased early mortality, and the overall mortality of such patients also seems to be lower than that observed in the general population.

## Supplementary Material

Supplemental Material

## Supplementary data

Tables 3 and 4 are available as supplementary data in the online version of this article, http://dx.doi.org/10.1080/17453674.2021.1963582
