# Supplementary material for: No increased mortality after total hip arthroplasty in patients with a history of pediatric hip disease: a matched, population-based cohort study on 4,043 patients
Source: Acta Orthop. 2021 Aug 16;92(6):673–7. doi: 10.1080/17453674.2021.1963582 (PMC8635541; doi:10.1080/17453674.2021.1963582)
Supplement: Supplemental Material [file IORT_A_1963582_SM8565.pdf]

Supplementary data

Table 3. 90-day mortality, adjusted for confounders alone or for confounders and matching variables (age, sex, and place of residence)

Item	Crude HR (95% CI)	Adj. HR (95% CI) [a]	Adj. HR (95% CI) [b]
All cases	0.8 (0.4–1.8)	0.7 (0.3–1.5)	0.9 (0.4–2.0)
LCPD [c]	–	–	–
SCFE [c]	–	–	–
DDH	0.9 (0.4–1.9)	0.7 (0.4–1.6)	0.9 (0.4–2.1)

[a] Adjustment for confounding factors, without matching variables.
[b] Adjustment for confounding factors and matching variables (age group, sex, and place of residence calculated as strata variable due to many variable levels (> 300)).
[c] No valid models could be fitted due to the scarcity of events.

Table 4. Overall mortality, adjusted for confounders and matching variables (age group, sex, and place of residence)

Item	Crude HR (95% CI)	Adj. HR (95% CI) [a]	Adj. HR (95% CI) [b]
All cases	0.9 (0.8–1.0)	0.8 (0.7–0.9)	0.8 (0.7–0.9)
LCPD	0.8 (0.6–1.3)	0.8 (0.6–1.3)	0.7 (0.4–1.3)
SCFE	0.9 (0.4–1.7)	0.9 (0.4–1.8)	2.1 (0.8–5.7)
DDH	0.9 (0.8–1.0)	0.8 (0.7–0.9)	0.8 (0.7–0.9)

[a] Adjustment for confounding factors, without matching variables.
[b] Adjustment for confounding factors and matching variables (age group, sex, and place of residence calculated as strata variable due to many variable levels (> 300)).
